# Comparative Analysis of Satellite DNA in the *Drosophila melanogaster* Species Complex

**DOI:** 10.1534/g3.116.035352

**Published:** 2016-12-22

**Authors:** Madhav Jagannathan, Natalie Warsinger-Pepe, George J. Watase, Yukiko M. Yamashita

**Affiliations:** * Howard Hughes Medical Institute, University of Michigan, Ann Arbor, Michigan 48109; † Life Sciences Institute, University of Michigan, Ann Arbor, Michigan 48109; ‡ Department of Molecular and Integrative Physiology, University of Michigan, Ann Arbor, Michigan 48109; § Department of Cell and Developmental Biology, University of Michigan, Ann Arbor, Michigan 48109

**Keywords:** *Drosophila*, evolution, fluorescent *in situ* hybridization, heterochromatin, satellite DNA

## Abstract

Satellite DNAs are highly repetitive sequences that account for the majority of constitutive heterochromatin in many eukaryotic genomes. It is widely recognized that sequences and locations of satellite DNAs are highly divergent even in closely related species, contributing to the hypothesis that satellite DNA differences may underlie speciation. However, due to its repetitive nature, the mapping of satellite DNAs has been mostly left out of recent genomics analyses, hampering the use of molecular genetics techniques to better understand their role in speciation and evolution. Satellite DNAs are most extensively and comprehensively mapped in *Drosophila melanogaster*, a species that is also an excellent model system with which to study speciation. Yet the lack of comprehensive knowledge regarding satellite DNA identity and location in its sibling species (*D. simulans*, *D. mauritiana*, and *D. sechellia*) has prevented the full utilization of *D. melanogaster* in studying speciation. To overcome this problem, we initiated the mapping of satellite DNAs on the genomes of the *D. melanogaster* species complex (*D. melanogaster*, *D. simulans*, *D. mauritiana*, and *D. sechellia*) using multi-color fluorescent *in situ* hybridization (FISH) probes. Our study confirms a striking divergence of satellite DNAs in the *D. melanogaster* species complex, even among the closely related species of the *D. simulans* clade (*D. simulans*, *D. mauritiana*, and *D. sechellia*), and suggests the presence of unidentified satellite sequences in these species.

Short tandem repetitive or “satellite” DNAs are abundant and conserved features of eukaryotic genomes. Although typically considered “junk” DNA due to a lack of protein coding potential, decades of study have implicated satellite DNA function in cellular processes such as kinetochore/centromere function, meiotic chromosome segregation, and X chromosome recognition (Dernburg *et al.* 1996; Karpen *et al.* 1996; Guenatri *et al.* 2004; Bouzinba-Segard *et al.* 2006; Usakin *et al.* 2007; Wong *et al.* 2007; Menon *et al.* 2014; Rosic *et al.* 2014). Moreover, aberrant transcription of satellite DNAs has been associated with human diseases such as cardiomyopathy and cancer, suggesting critical importance of the regulation of this underappreciated component of eukaryotic genomes (Gaubatz and Cutler 1990; Feber *et al.* 2011; Ting *et al.* 2011; Haider *et al.* 2012). Yet, other than these examples, the functions of the majority of satellite DNAs remain obscure.

The common fruit fly, *Drosophila melanogaster*, is an excellent model system with which to study satellite DNAs. Approximately 21% of the *D. melanogaster* genome is comprised of satellite DNA (Lohe and Brutlag 1986) and extensive efforts have mapped the location of 15 unique repeats on *D. melanogaster* chromosomes (Waring and Pollack 1987; Bonaccorsi and Lohe 1991; Abad *et al.* 1992; Lohe *et al.* 1993; Dernburg *et al.* 1996). Efforts to identify satellite repeats and map them onto chromosomes have been made in many species including *Abracris flavolineata* (Bueno *et al.* 2013), *Aegilops geniculate* and wheat (Koo *et al.* 2016), various *Arabidopsis* species (Kamm *et al.* 1995; Ito *et al.* 2007; Kawabe and Charlesworth 2007), maize (Lamb *et al.* 2007), *Turritis glabra* (Kawabe and Nasuda 2006), rodent species including *Phodopus sungorus* (Paco *et al.* 2014), *Cricetus cricetus* and *Microtus arvalis* (Louzada *et al.* 2015), *Xenopus* (Schmid and Steinlein 2015), and human (Altemose *et al.* 2014), revealing general patterns of centromeric, pericentromeric, and telomeric satellite distribution. The rich history of *D. melanogaster* genetics has resulted in the comprehensive identification and mapping of satellite DNA to individual chromosomes; *D. melanogaster* remains the only species with this resolution (Waring and Pollack 1987; Bonaccorsi and Lohe 1991; Abad *et al.* 1992; Lohe *et al.* 1993; Dernburg *et al.* 1996). Even in *Drosophila* sibling species such as *D. simulans*, *D. mauritiana*, and *D. sechellia* (together called the *D. simulans* clade), satellite composition and chromosome location has only been partially examined (Lohe and Brutlag 1987; Larracuente 2014).

Interestingly, it has been shown that even closely related species display significant divergence in the abundance and sequence of individual satellite DNA repeats (Lohe and Brutlag 1987; Lohe and Roberts 2000; Bosco *et al.* 2007). These observations led to the hypothesis that rapid divergence of satellite DNA may play an important role in speciation by causing reproductive isolation between closely related species (Yunis and Yasmineh 1971; Gatti *et al.* 1976). In support of this idea, it was shown that a satellite DNA on the *D. melanogaster* X chromosome (*i.e.*, 359 bp repeats) causes hybrid incompatibility when crossed with its closest sibling species, *D. simulans* (Sawamura *et al.* 1993; Ferree and Barbash 2009). However, a lack of information regarding satellite DNAs in other species hinders efforts to identify whether there are more instances of hybrid incompatibility caused by satellite DNA among closely related species.

In this study, we have used FISH to map known *Drosophila* satellite DNA repeats on the mitotic chromosomes of the sibling species *D. melanogaster*, *D. simulans*, *D. mauritiana*, and *D. sechellia* (collectively categorized as “the *D. melanogaster* species complex”). We reveal a remarkable divergence in the abundance and location of specific satellite DNA repeats in these closely related sibling species, and provide this information as a resource for future work on *Drosophila* chromosome biology and speciation.

## Materials and Methods

### Drosophila strains and fly husbandry

All fly stocks were raised on standard Bloomington medium at 25°, and male third instar wandering larvae were used. For better chromosome squash, larvae cultured at 18° were used. The following fly stocks were used: *D. melanogaster* yw, *D. simulans w^501^* (DSSC#14021-0251.195), *D. sechellia* *w*^*1*^ (DSSC#14021-0248.30), and *D. mauritiana* *w^1^* (DSSC#14021-0241.60).

### Larval brain squash, chromosome FISH, and microscopy

We adapted a simple FISH protocol against squashed chromosomes published by Larracuente and Ferree (2015) with small modifications. Briefly, male third instar wandering larvae were collected and brains were dissected in PBS. Larval brains were fixed in 25 µl of acetic acid: 4% formaldehyde in PBS (45%:55%) for 4 min on Sigmacote-coated coverslips (Sigma: SL2 SIGMA). The whole sample was quickly applied to a clean Superfrost plus slide and the sample was manually squashed via thumb/stamp over coverslip, over sample, on top of the slide. The slide/coverslip/sample was immediately submerged in liquid nitrogen until it stopped boiling. Slides were removed from liquid nitrogen and coverslips were quickly flicked off the slide with a razor blade. Slides were then washed in 100% ethanol at room temperature for 5 min then dried in a dark, dust-free location.

Hybridization was performed in 50% formamide, 10% dextran sulfate, 2 × SSC buffer, 0.5 µM of each probe, and up to 20 µl of diH_2_O. Hybridization buffer was added to the samples and covered with a coverslip. Slides were heated at 95° for 5 min, cooled briefly, wrapped in parafilm, and incubated in a humid chamber in the dark overnight at room temperature. Coverslips were removed and slides were washed three times for 15 min in 0.1 × SSC, removed of excess buffer, and mounted in Vectashield mounting medium containing DAPI.

Images were taken using an upright Leica TCS SP8 confocal microscope with a 63 × oil immersion objective (NA = 1.4) and processed using Adobe Photoshop software. Images were modified solely for the purpose of clarity. Modified images were not quantified.

Detailed sequences of probes used that are not provided in Table 1 are as follows: 359 (part of 359 bp unit, antisense direction), 5′-AGGATTTAGGGAAATTAATTTTTGGATCAATTTTCGCATTTTTTGTAAG-3′; 372 (part of 372 bp unit), 5′-TATTTTGATCAAAACATTGAAAATAATGGCCCAAATATGGAATGTCATACCTCGTTGAGTTTGTTTTTTA-3′; IGS (part of 240 bp unit), 5′-AGTGAAAAATGTTGAAATATTCCCATATTCTCTAAGTATTATAGAGAAAAGCCATTTTAGTGAATGGA-3′; dodeca, 5′-ACCGAGTACGGGACCGAGTACGGGACCAGTACGGGACCAGTACGGG-3′; 260 (part of 260 bp unit), 5′-CATATTTGCAAATTTTGATGAATGCGAAAATTAACC-3′.

**Table 1:** Combination of probes used for FISH mapping

Combination	488 (Green)	Red (Cy3)	Blue (Cy5)
C1	(AATAT)_6_	(AATAG)_6_	359
C2	(AATAGAC)_6_	(AATAC)_6_	(AATAAAC)_6_
C3	IGS	(AAGAG)_6_	Prod: (AATAACATAG)_3_
C4	IGS	372	359
C5	(AAGAG)_6_	Dodeca	(AACAC)_6_
C6	(AATAT)_6_	(GAACAGAACATGTTC)_2_	(AACAAAC)_5_
C7	(AATAT)_6_	(AAGAC)_6_	(AAAAC)_6_
C8	(AAGAG)_6_	(AAGAC)_6_	(AAGAGAG)_5_
C9	(AATAT)_6_	(GAACAGAACATGTTC)_2_	359
C10	(AATAT)_6_	(GAACAGAACATGTTC)_2_	Dodeca
C11	(AATAT)_6_	(TAGA)_8_	260

See *Materials and Methods* for the sequence details of the probes for 359, 372, 260, IGS, and dodeca.

### Data availability

The authors state that all data necessary for confirming the conclusions presented in the article are represented fully within the article.

## Results

Based on previous studies (Simeone *et al.* 1985; Lohe and Brutlag 1987; Waring and Pollack 1987; Lohe and Roberts 1990; Abad *et al.* 1992, 2000; Carmena *et al.* 1993; Lohe *et al.* 1993; Hoskins *et al.* 2015), we designed 17 probes that are known to exist in *D. melanogaster* (some of which are also known to exist in *D. simulans*), and two probes that are known to exist in *D. simulans* but are supposedly absent in *D. melanogaster* (Table 1). We used these 19 probes in 11 different combinations (C1–11, Table 1), with each combination consisting of Alexa488-, Cy3-, and Cy5-conjugated probes. We then performed FISH on the mitotic chromosomes of squashed larval brains from *D. melanogaster*, *D. simulans*, *D. mauritiana*, and *D. sechellia* using a method described previously (Larracuente and Ferree 2015). We selected male third instar larvae based on their gonad morphology such that the mitotic chromosome spread would always contain a Y chromosome.

**Figure 1:**
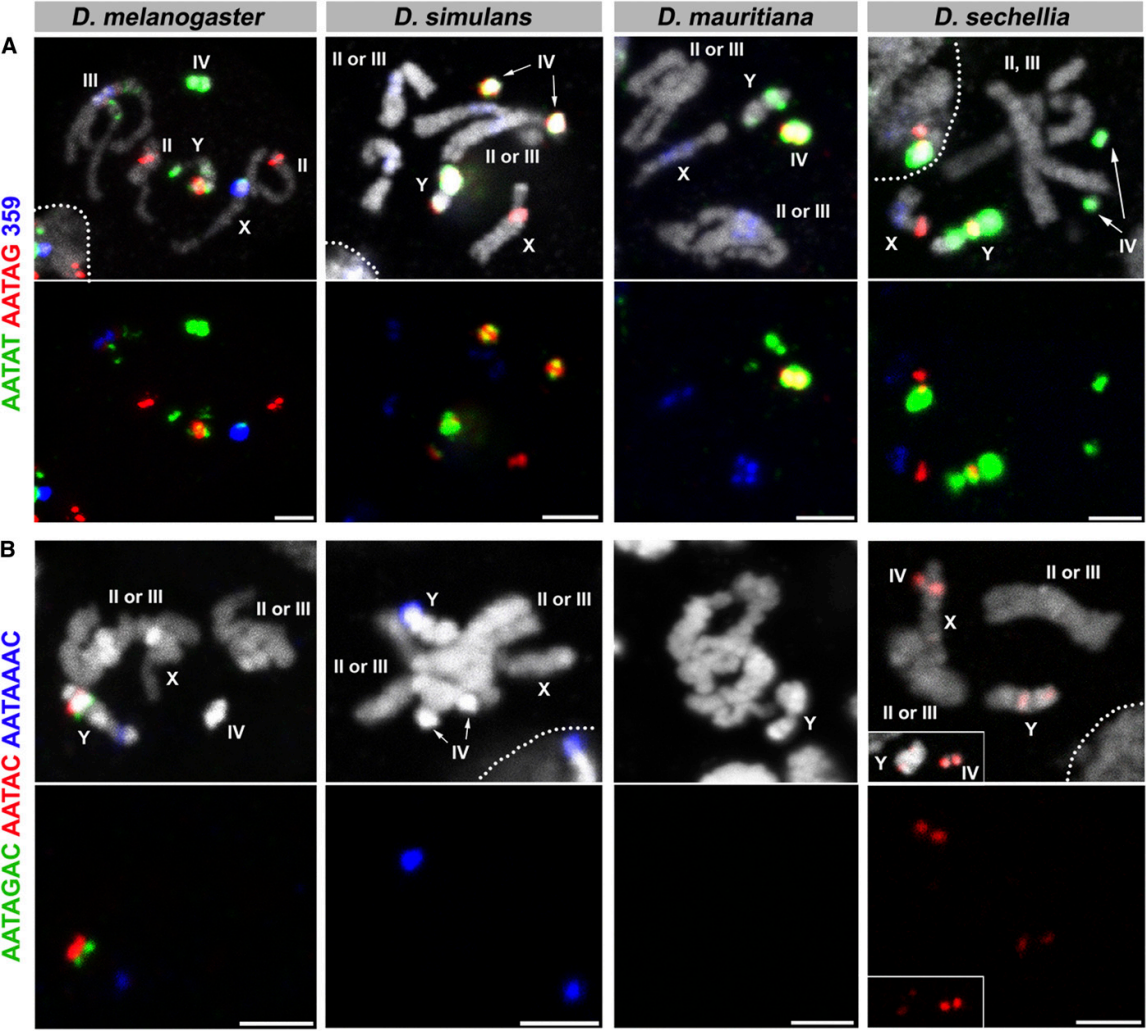
FISH on neuroblast chromosome spread from *D. melanogaster*, *D. simulans*, *D. mauritiana*, and *D. sechellia*. (A) C1 probes and (B) C2 probes. Probe sequences are indicated by the colored text. The top panels show three probes combined with DAPI, and the bottom panels show only probe hybridization signals. Bar, 2.5 µm. C, combination; DAPI, 4’,6-diamidino-2-phenylindole; FISH, fluorescent *in situ* hybridization.

**Figure 2:**
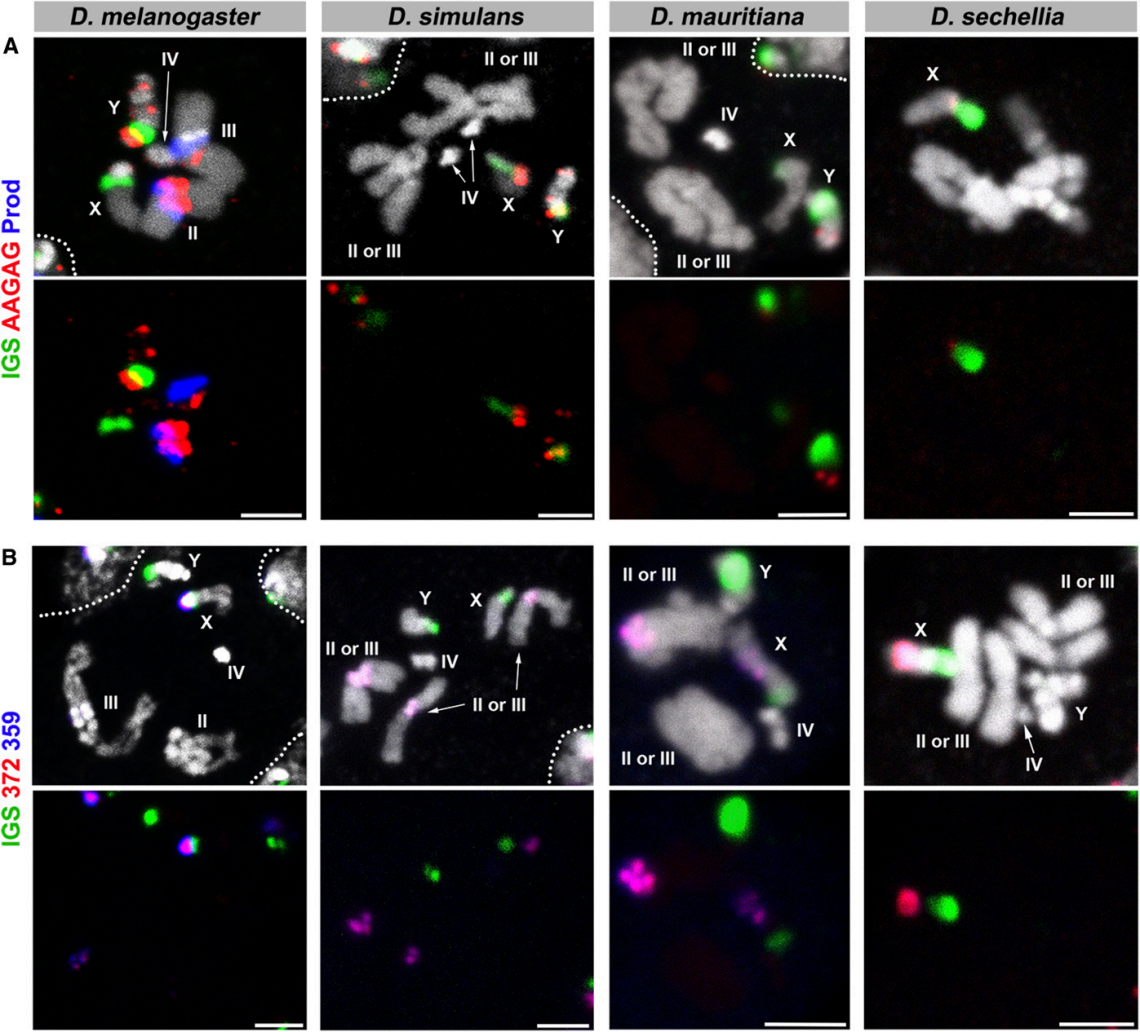
FISH on neuroblast chromosome spread from *D. melanogaster*, *D. simulans*, *D. mauritiana*, and *D. sechellia*. (A) C3 probes and (B) C4 probes. Probe sequences are indicated by the colored text. The top panels show three probes combined with DAPI, and the bottom panels show only probe hybridization signals. Bar, 2.5 µm. C, combination; DAPI, 4’,6-diamidino-2-phenylindole; FISH, fluorescent *in situ* hybridization.

**Figure 3:**
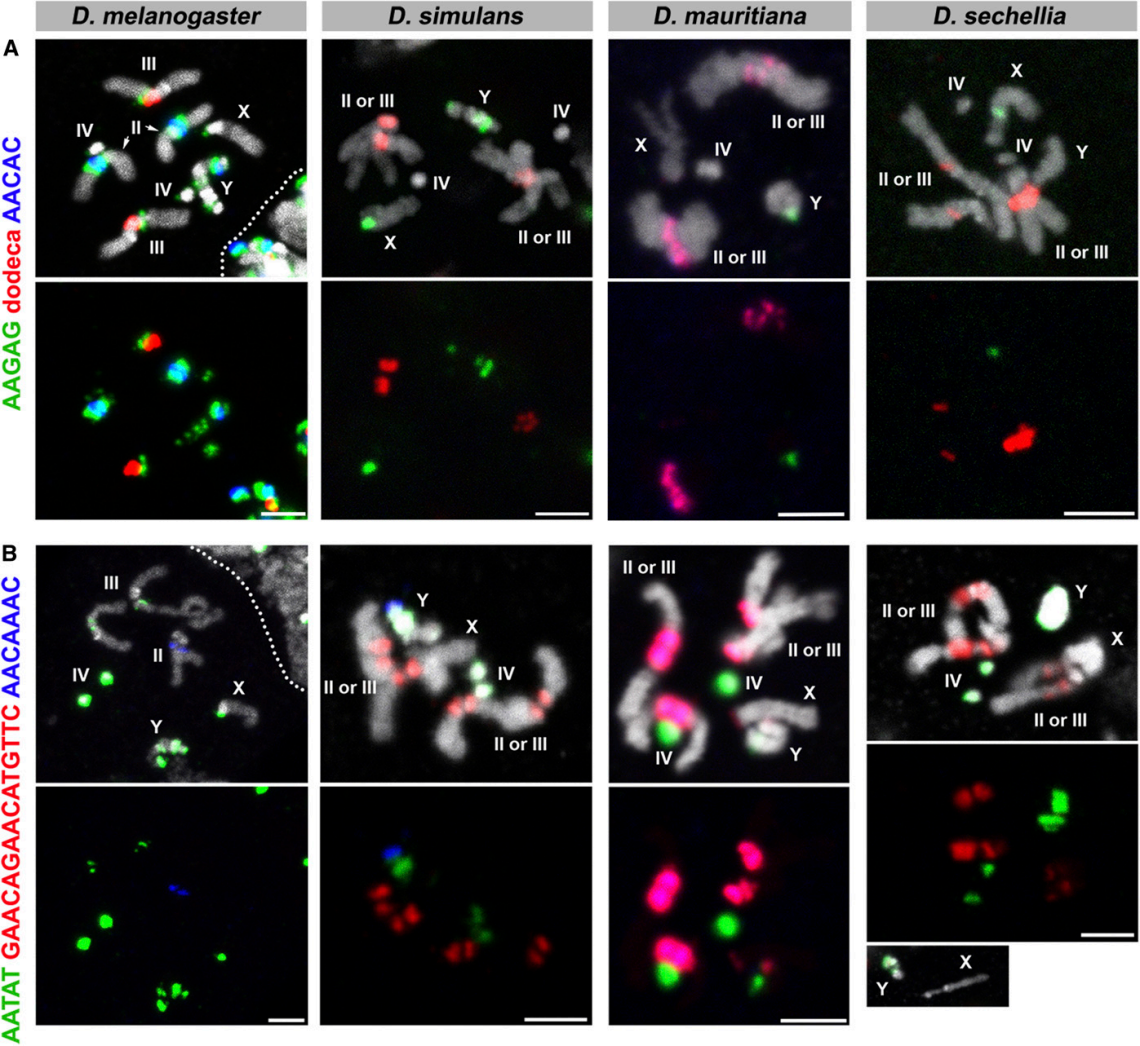
FISH on neuroblast chromosome spread from *D. melanogaster*, *D. simulans*, *D. mauritiana*, and *D. sechellia*. (A) C5 probes and (B) C6 probes. Probe sequences are indicated by the colored text. The top panels show three probes combined with DAPI, and the bottom panels show only probe hybridization signals. Bar, 2.5 µm. C, combination; DAPI, 4’,6-diamidino-2-phenylindole; FISH, fluorescent *in situ* hybridization.

**Figure 4:**
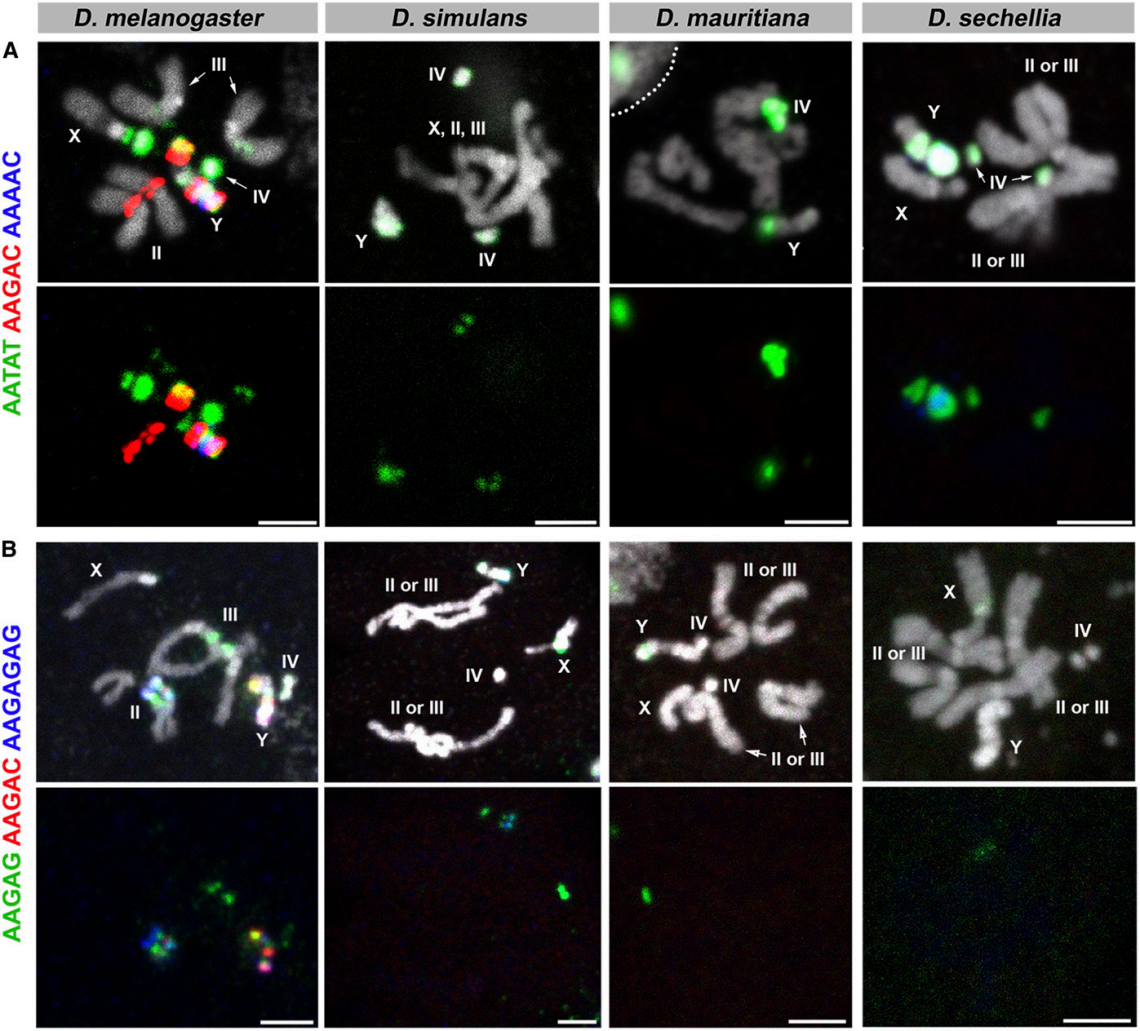
FISH on neuroblast chromosome spread from *D. melanogaster*, *D. simulans*, *D. mauritiana*, and *D. sechellia*. (A) C7 probes and (B) C8 probes. Probe sequences are indicated by the colored text. The top panels show three probes combined with DAPI, and the bottom panels show only probe hybridization signals. Bar, 2.5 µm. C, combination; DAPI, 4’,6-diamidino-2-phenylindole; FISH, fluorescent *in situ* hybridization.

**Figure 5:**
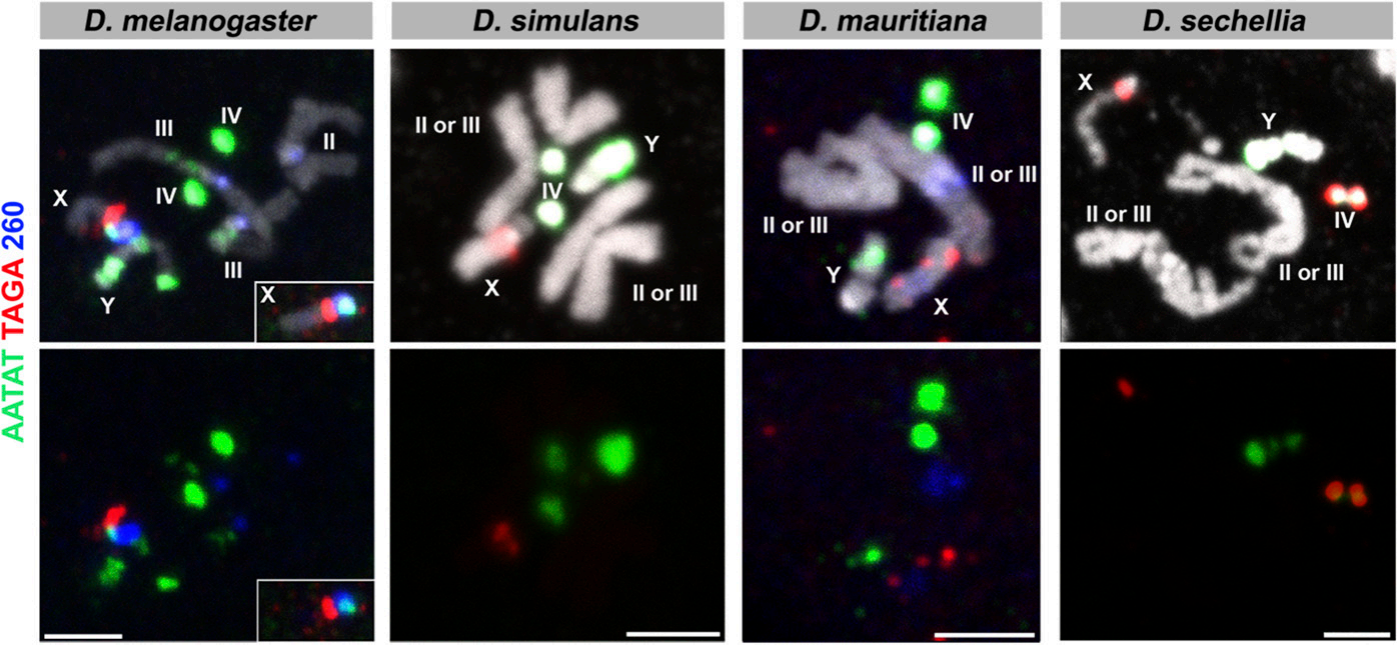
FISH on neuroblast chromosome spread from *D. melanogaster*, *D. simulans*, *D. mauritiana*, and *D. sechellia*. C11 probes were used. Probe sequences are indicated by the colored text. The top panels show three probes combined with DAPI, and the bottom panels show only probe hybridization signals. Bar, 2.5 µm. C, combination; DAPI, 4’,6-diamidino-2-phenylindole; FISH, fluorescent *in situ* hybridization.

The results are shown in Figure 1, Figure 2, Figure 3, Figure 4, and Figure 5, and the chromosomal location(s) of each satellite DNA repeat in all four species are summarized in Table 2 and Table 3. We observed a dramatic divergence of satellite repeat abundance and distribution that clearly demarcates the chromosomes of these closely related species. It should be noted that the probe used for 372 bp repeats perfectly colocalized with the 359 bp probe on the mitotic chromosomes in all four species, suggesting that these two probes do not differentiate between the 359 bp satellite and the 372 bp repeat sequences, as well as other derivatives such as 353 and 356 bp repeats in *D. melanogaster* (Losada and Villasante 1996; Abad *et al.* 2000), and 360 family sequences in the *D. simulans* clade (Strachan *et al.* 1985). Indeed, our 70-bp-long probe designed for 372 bp repeats shows 74% identity to the 359 bp satellite consensus sequence. One puzzling observation is that the 372 bp repeats were originally identified as middle repetitive elements dispersed along the X chromosome euchromatin in *D. melanogaster* (Waring and Pollack 1987). Later, it was found that the 372 bp repeats are a part of the 1.688 g/cm^3^ class of satellite DNAs, which is present at numerous locations throughout the X chromosome euchromatin (DiBartolomeis *et al.* 1992). Our mitotic chromosome FISH did not detect hybridization of 372 or 359 bp probes to these euchromatic regions. This might be due to the fact that euchromatic loci of the 1.688 g/cm^3^ class of satellite DNAs (including 372 bp repeats) are below the detection limit of our FISH technique (DiBartolomeis *et al.* 1992; Kuhn *et al.* 2012). In addition, the detection of such low abundance sequences in euchromatic regions might require polytenization, where the euchromatic 372 bp repeat was originally identified (Waring and Pollack 1987). We additionally designed a 260 bp probe, as neither of our 359 or 372 bp probes was expected to hybridize to 260 bp repeats. This probe still cross-hybridized to 359/372 bp repeats, but successfully detected an additional locus (Figure 5), likely representing *bona fide* 260 bp repeats on the *D. melanogaster* second chromosome as reported previously (albeit at a low signal intensity) (Abad *et al.* 2000). In *D. mauritiana*, the 260 bp probe hybridized to the same chromosomal location as 359/372 bp repeats, suggesting this might also represent cross-hybridization. However, in *D. simulans* and *D. sechellia*, the 260 bp repeat probe did not show any signal, suggesting either that (1) *D. simulans* and *D. sechellia* 359/372 bp repeats are more diverged in the region that is covered by our 260 bp repeat probe or that (2) cross-hybridization of the 260 bp probe to 359/372 bp repeats is very weak and slight differences in hybridization conditions yield different results.

**Table 2:** Location of the satellite DNAs on the chromosomes of the *D. melanogaster* species complex

	*D. melanogaster*	*D. simulans*	*D. mauritiana*	*D. sechellia*
(AATAT)_n_	Y, X, III, IV	Y, IV	Y, IV	Y, IV
(AATAG)_n_	Y, II, III*^a^*	Y, X, IV	IV	Y, X
359/372	X*^b^*, III*^b^*	II*^b^*, III*^b^*	X*^b^*, II or III*^b^*	X*^b^*
260	X*^b^*, III*^b^*, II	None	X*^b^*, II or III*^b^*	None
(AATAGAC)_n_	Y	None	None	None
(AATAC)_n_	Y	None	None	Y, IV
(AATAAAC)_n_	Y	Y	None	None
IGS	X, Y	X, Y*^c^*	X, Y	X (not on Y*^c^*^,^*^d^*)
(AAGAG)_n_	X, Y, II, III, IV	X, Y	Y	X
Prod	II, III	None	None	None
Dodeca	Y*^e^*, III	II, III	II, III	II, III
(AACAC)_n_	Y, II	None	II*^a^*, III*^a^*	None
(GAACAGAACATGTTC)_n_	None	II, III	II, III, X*^a^*, Y*^a^*	II, III
(AACAAAC)_n_	II*^a^*	Y	II*^a^*, III*^a^*	None
(AAGAC)_n_	Y, II	None	None	None
(AAAAC)_n_	Y	None	None	Y
(AAGAGAG)_n_	Y, II	Y	None	None
(TAGA)_n_	X	X	X	X, IV

rDNA, ribosomal DNA.

a Indicates the sequences that are obviously low in abundance.

b Likely cross-hybridization among 359 bp, 372 bp, 260 bp, and other related repeats.

c Without rDNA.

d Roy *et al.* (2005) reports the presence of rDNA (using Py12 and 240 bp probes) on *D. sechellia* Y, although much weaker than X rDNA. This may be because our IGS-240 probe does not cover the entire length of the 240 bp sequence, and our probe sequence does not hybridize well to *D. sechellia* IGS.

e Yadlapalli and Yamashita (2013) described its presence on Y but this chromosome spread did not find dodeca on Y, possibly due to differences in hybridization conditions or strain.

**Table 3:** Satellite composition of each chromosome in the *D. melanogaster* species complex

	*D. melanogaster*	*D. simulans*	*D. mauritiana*	*D. sechellia*
X	AATAT			
	359/372/260		359/372/260	359/372
	IGS	IGS	IGS	IGS
	AAGAG	AAGAG		AAGAG
		AATAG		AATAG
			GAACAGAACATGTTC	
	TAGA	TAGA	TAGA	TAGA
Y	AATAT	AATAT	AATAT	AATAT
	AAGAC			
	AATAC			AATAC
	AATAG	AATAG		AATAG
	AATAGAC			
	AATAAAC	AATAAAC		
	IGS	IGS	IGS	
	AAGAG	AAGAG	AAGAG	
	AACAC			
	AAAAC			AAAAC
	AAGAGAG	AAGAGAG		
		AACAAAC		
			GAACAGAACATGTTC*^a^*	
II	AATAG			
	AAGAG			
	Prod			
	AACAC		AACAC	
	AAGAC			
	AACAAAC		AACAAAC	
	AAGAGAG			
	260	359/372	359/372/260 (only II or III)	
		Dodeca	Dodeca	Dodeca
		GAACAGAACATGTTC	GAACAGAACATGTTC	GAACAGAACATGTTC
III	AATAT			
	AATAG			
	AAGAG			
	359/372/260	359/372	359/372/260 (only II or III)	
	Prod			
	Dodeca	Dodeca	Dodeca	Dodeca
			AACAAAC	
		GAACAGAACATGTTC	GAACAGAACATGTTC	GAACAGAACATGTTC
			AACAC	
IV	AATAT	AATAT	AATAT	AATAT
	AAGAG			
		AATAG	AATAG	
				AATAC
				TAGA

a Indicates a sequence that is obviously low in abundance.

While two *D. simulans*-specific satellites (AACAAAC and GAACAGAACATGTTC) had been previously identified by cesium chloride centrifugation (Lohe and Brutlag 1987), their chromosomal locations were unknown. Here, we map their locations on the *D. simulans* chromosomes (Figure 3, Table 2, and Table 3) and extend this mapping to both *D. mauritiana* and *D. sechellia*. The GAACAGAACATGTTC repeat is completely absent in *D. melanogaster* and is predominantly present on the autosomes of *D. simulans*, *D. mauritiana*, and *D. sechellia*. On the other hand, the AACAAAC satellite repeat is specifically present on the *D. simulans* Y chromosome and is absent from *D. mauritiana* and *D. sechellia*. Although AACAAAC was reported to be absent in *D. melanogaster*, we observed probe hybridization on the *D. melanogaster* second chromosome, albeit at a low level. It is possible that a slightly diverged sequence on the *D. melanogaster* chromosome is still able to hybridize to the AACAAAC probe. Alternatively, the *D. melanogaster* genome could have acquired/amplified this satellite repeat in the time between the previous report and this study (∼30 yr).

**Figure 6:**
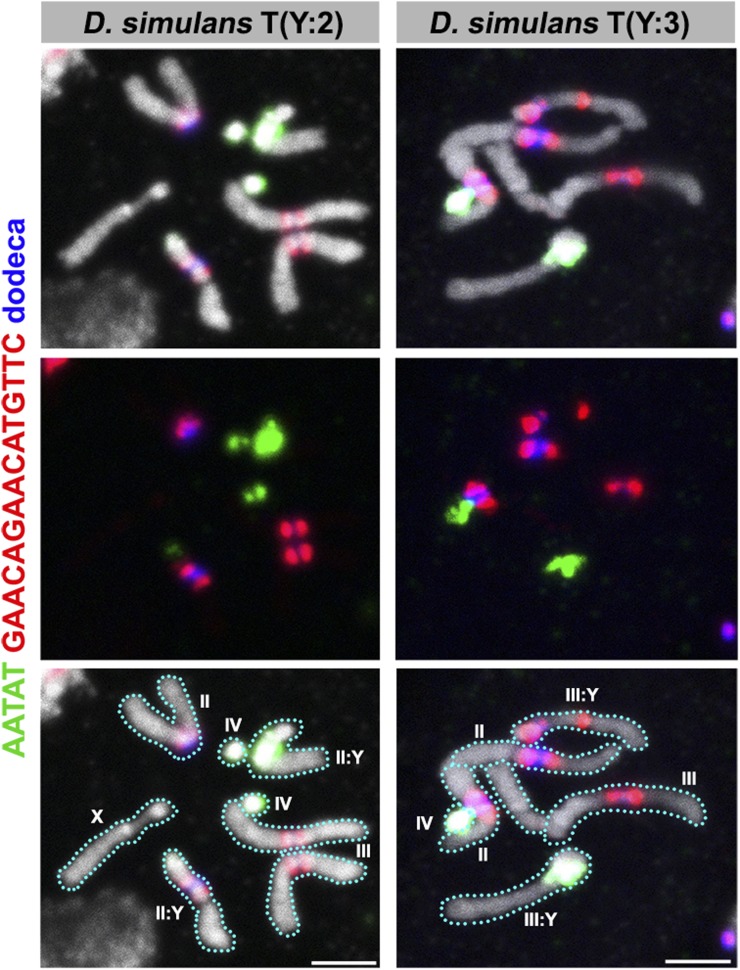
FISH on neuroblast chromosome spread from *D. simulans* T(Y:2) and T(Y:3) translocation strains. C10 probes were used. Probe sequences are indicated by the colored text. The top panels show three probes combined with DAPI, the middle panels show only probe hybridization signals and the bottom panels indicate the interpretation. Bar, 2.5 µm. C, combination; DAPI, 4’,6-diamidino-2-phenylindole; FISH, fluorescent *in situ* hybridization.

An important aspect of the satellite DNA maps in *D. melanogaster* is the knowledge of chromosome-specific satellite sequences, a feature that has facilitated the study of chromosome-specific behaviors (Dernburg *et al.* 1996; Joyce *et al.* 2012, 2013; Christophorou *et al.* 2013). Although our FISH experiments revealed differences in the contents of satellite DNA repeats on the autosomes of the *D. simulans* clade, it is nevertheless difficult to determine which chromosome (second or third) corresponds to which satellite composition, as these two autosomes are morphologically indistinguishable in all species. We have attempted to overcome this issue in *D. simulans* using two stocks, Dsim\T(Y;2) and Dsim\T(Y;3), where a translocation event between the Y and second or third chromosomes allows us to unambiguously distinguish the second and third chromosomes. By using a probe combination that focuses on *D. simulans* autosomes (C10, Table 1), we observed these translocation stocks of *D. simulans*. Our data show that the majority of the dodeca satellite repeats are present on the *D. simulans* second chromosome (Figure 6). Considering the fact that the dodeca satellite repeat is present on the third chromosome in *D. melanogaster*, it is clear that each autosome does not have “signature” sequences (*i.e.*, chromosome-specific sequences) that are conserved among the four species examined in this study. Due to the lack of translocation stocks in *D. mauritiana* and *D. sechellia*, the identity of the second and third chromosomes with respect to satellite DNA composition remains unclear in these species.

**Figure 7:**
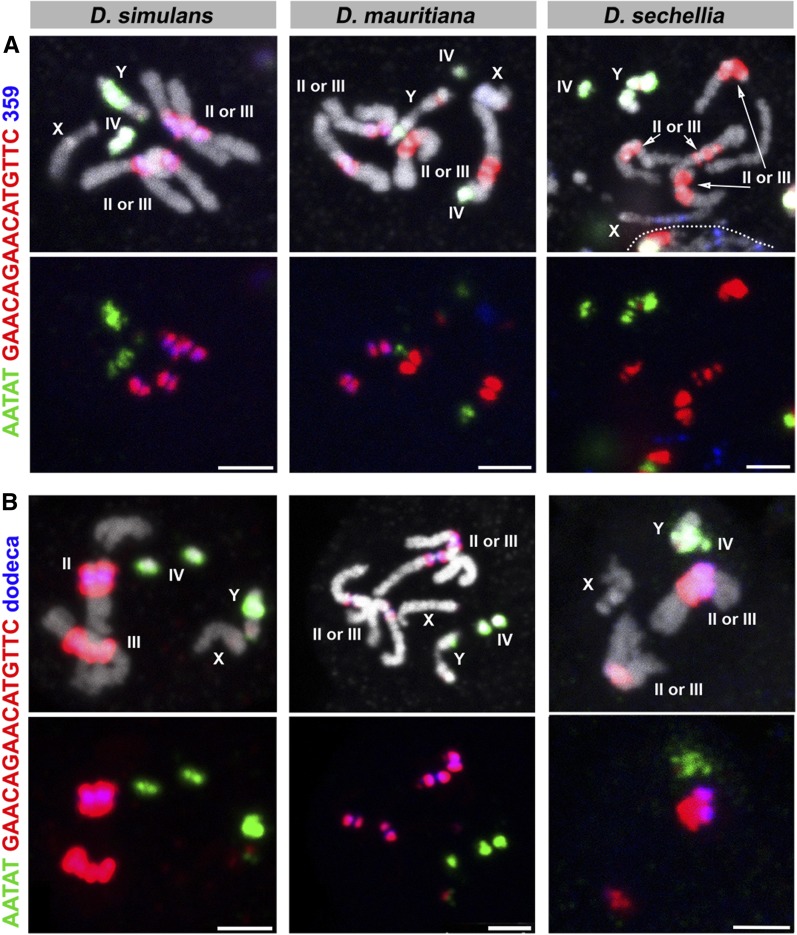
FISH on neuroblast chromosome spread from *D. simulans*, *D. mauritiana*, and *D. sechellia*. (A) C9 probes and (B) C10 probes. Probe sequences are indicated by the colored text. The top panels show three probes combined with DAPI, and the bottom panels show only probe hybridization signals. Bar, 2.5 µm. C, combination; DAPI, 4’,6-diamidino-2-phenylindole; FISH, fluorescent *in situ* hybridization.

Finally, we observed that the GAACAGAACATGTTC and 359/372 satellite repeats were asymmetrically distributed between the *D. mauritiana* second and third chromosomes (Figure 2, C4 and Figure 3, C6); that is, one chromosome (second or third) contained more GAACAGAACATGTTC satellites than the other, and similarly, one chromosome contained more 359/372 repeats than the other chromosome. Also, we observed a similar asymmetric distribution of the GAACAGAACATGTTC and the dodeca satellite repeats on the *D. sechellia* second and third chromosomes (Figure 3, C5 and C6). To determine the relationship between these satellite repeats on the autosomes of *D. mauritiana* and *D. sechellia*, we used two probe combinations (C9 and C10) (Figure 7 and Table 1). This revealed that the abundances of 359 bp and GAACAGAACATGTTC satellite repeats are inversely correlated in *D. mauritiana*; the autosome that contains more 359 bp repeats contains less GAACAGAACATGTTC compared to the other autosome. Also, in *D. mauritiana*, the abundances of GAACAGAACATGTTC and dodeca satellite repeats are positively correlated. In *D. sechellia*, the abundance of GAACAGAACATGTTC and dodeca are positively correlated; the autosome that contained more GAACAGAACATGTTC also contained more dodeca compared to the other autosome.

## Discussion

*D. melanogaster* is one of the few species where satellite DNA has most comprehensively been mapped (Lohe *et al.* 1993). In previous studies, the mapping effort was carried out by the use of radioactive probes, allowing precise quantitation of satellite DNA repeats. In addition, the extensive and unique set of chromosomal aberrations in this historical model organism facilitated the mapping of these repeats onto specific chromosomal loci. However, these mapping experiments cannot be extended to the sibling species of *D. melanogaster* due to the lack of a similar set of chromosomal aberrations. Therefore, other approaches are required to resolve the relationship of multiple satellite repeats on the chromosomes of sibling species.

Here, by using multi-color FISH, we have mapped previously identified satellite DNAs onto the genomes of the *D. melanogaster* species complex. To our knowledge, this is the most comprehensive mapping of satellite DNAs across all the species comprising the *D. melanogaster* species complex to date.

We estimate that the detection limit of our method is between 110 and 140 kb. By comparing our FISH results with the previous mapping effort by Lohe *et al.* (1993), which used ^3^H-labeled radioactive probes and is more accurate in quantification, the detection limit of our method can be inferred (Table 4). Except for (AATAG)_n_ on the III, which was estimated to be 30 kb and detected with our method, all satellite repeats that were estimated below 110 kb by Lohe *et al.* (1993) were not detected with our method, whereas all satellite repeats that were estimated above 140 kb by Lohe *et al.* (1993) were detectable with our method (Table 4). Therefore, it is possible that the satellite repeats that exist below 140 kb in the *D. simulans* clade species were missed in this study.

**Table 4:** Estimating the detection limit of FISH method used in this study

Satellite	X	Y	II	III	IV
(AATAT)_n_	**600**	**5800**	10	**630**	**2700**
(AATAG)_n_	8.1	**310**	**200**	**30***^a^*	78
(AATAC)_n_	0	**3500**	0	0	0
(AAAAC)_n_	0	**400**	0	0	0
(AAGAC)_n_	81	**8500**	**1800**	110	0
(AAGAG)_n_	**1200**	**7200**	**5500**	**1100**	**170**
(AATAAAC)_n_	0	**1600**	0	0	0
(AATAGAC)_n_	0	**1600**	0	0	0
(AAGAGAG)_n_	**270**	**1800**	**1700**	**140**	100
(AATAACATAG)_n_	0	0	**1900**	**1600**	0
359 bp	**11000**	0	0	0	0

The numbers indicate the amounts (kilobases) of satellite repeats in the *D. melanogaster* genome estimated by Lohe *et al.* (1993). Bold numbers indicate the satellite repeats that were successfully detected with our FISH method. FISH, fluorescent *in situ* hybridization.

a Except for 30 kb of (AATAG)_n_, which was detected on the third chromosome, the detection limit with our method is likely between 110 and 140 kb [110 kb of (AAGAC)_n_ being undetectable and 140 kb of (AAGAGAG)_n_ being detectable].

Our work provides a few critical insights into the arrangement and evolution of satellite DNA and reveals the need for future mapping efforts in these species.

### Satellite DNA divergence in the D. melanogaster species complex

Lohe and Brutlag (1987) suggested that after *D. melanogaster* and *D. simulans* diverged from a common ancestor, the satellite DNA sequences between both of these species remained similar in sequence, although different in abundance. Although quantification is not possible with our method of analysis, our study confirms that some satellite DNAs are visibly different in abundance between these species (*e.g.*, 359 bp, AATAAAC, AATAC, AAGAG, AACAC, AAGAC, AAGAGAG, GAACAGAACATGTTC, and prod satellite). Furthermore, our mapping in this study reveals that *D. melanogaster* and *D. simulans* differ from each other in the patterning and location of these satellite DNA repeats. Among four species of the *D. melanogaster* species complex, a few satellite DNA repeats are found to be absent in one or more species, suggesting species specificity of certain satellites. Overall, it is clear that satellite DNA distribution/composition in *D. melanogaster* is most distant within the *D. melanogaster* species complex, whereas those of three species in the *D. simulans* clade (*D. simulans*, *D. mauritiana*, and *D. sechellia*) are much closer to each other, just like their evolutionary relationship. However, it is apparent that there are drastic differences in satellite DNA contents even among species of the *D. simulans* clade. This suggests that changes in satellite DNA sequence/patterning occurs rapidly over the short evolutionary timespan that separates sibling species, as suggested by Lohe and Brutlag (1987) and Hey and Kliman (1993).

In addition to distinct composition of satellite DNA repeats in these species, multi-color FISH revealed that satellites that are present in distinct locations in one species are overlapping in another species. For instance, the dodeca satellite and AACAC are present at distinct loci on separate autosomes in *D. melanogaster*, but completely overlap in *D. mauritiana* (Figure 3, C5). In another example, GAACAGAACATGTTC and AACAAAC overlap in *D. mauritiana* but are separate in *D. simulans*, where GAACAGAACATGTTC remains on the autosomes and AACAAAC is only present on the Y chromosome (Figure 3, C6). Overlapping signals likely reflect that those satellite sequences are interspersed among each other without forming large blocks of repeats composed of a single sequence. The fact that the repeats are interspersed in one species, but completely separate in another species, indicates that satellite evolution cannot be explained by a single simple translocation event that cuts/copies and pastes a large segment of a chromosome to another. Instead, multiple events of one or many mechanisms must occur to account for the diverse patterning and abundance of satellite DNA repeats in closely related species. Potential underlying mechanisms include transposition and nonhomologous recombination among satellites (Ugarkovic and Plohl 2002; Plohl *et al.* 2012).

### Possible presence of unidentified satellite DNA sequences in the D. simulans clade species

In this study, we mainly utilized satellite DNA probes that were previously identified in *D. melanogaster* and performed comparative mapping in the entire *D. melanogaster* species complex. This leaves the possibility that there are unidentified satellite repeats in *D. simulans* clade species that were left out of our mapping effort. Although we were able to cover the majority of the Y chromosomes of *D. melanogaster*, *D. simulans*, and *D. sechellia* using our satellite DNA probes, only a minor portion of the *D. mauritiana* Y chromosome was visualized by our probe set. As it was shown that the *D. mauritiana* Y chromosome contains a large amount of transposons (*HeT-A* and *TART*) (Berloco *et al.* 2005), it is possible that these transposons are the major constituent of Y chromosome heterochromatin in *D. mauritiana*. It will be of future interest to identify unknown satellite repeats in *D. simulans* clade species. It is possible that those unidentified satellites belong to the classes of “complex repeats,” such as 180 and 500 bp repeats, as indicated previously (Strachan *et al.* 1982).

In summary, our comparative mapping of satellite DNA using multi-color FISH probes has confirmed the highly divergent nature of satellite DNAs among the *D. melanogaster* species complex, and identified species-specific and chromosome-specific satellites. We hope that this study will serve as a resource for future work on chromosome biology and speciation in these model organisms.
